# OAM multiplication operator enabled holographic multiplexing

**DOI:** 10.1038/s41377-025-02107-2

**Published:** 2026-01-02

**Authors:** Feiyang Shen, Zhengyang Mao, Weiwen Fan, Jiangwei Wu, Zhifan Fang, Haigang Liu, Xianfeng Chen, Yong Zhang, Yuping Chen

**Affiliations:** 1 State Key Laboratory of Photonics and Communications, School of Physics and Astronomy, Shanghai Jiao Tong University, Shanghai, China; 2 National Laboratory of Solid State Microstructures, College of Engineering and Applied Sciences, Nanjing University, Nanjing, China; 3 Collaborative Innovation Center of Light Manipulations and Applications, Shandong Normal University, Jinan, China

**Keywords:** Optical data storage, Imaging and sensing

## Abstract

Holography has emerged as a vital platform for three-dimensional displays, optical encryption, and photonic information processing, leveraging diverse physical dimensions of light such as wavelength, polarization, and orbital angular momentum (OAM) to expand multiplexing capacity. However, the exhaustive utilization of these intrinsic degrees of freedom has saturated the parameter space for holographic encoding, leaving no room for further scalability. Here, we demonstrate an OAM multiplication operator enabled holographic multiplexing. We engineer the operator-specific hologram that selectively responds to the predefined operator pathway. Subsequent validation of orthogonality between distinct operator pathways ensures the multiplexing ability, thereby enabling the parallel encoding of multiple holographic images. In the experiment, we have successfully demonstrated a ninefold capacity enhancement over conventional OAM holography and a 2-bit operator-multiplexed hologram for high-security optical encryption. This work introduces operators as a synthetic dimension beyond light’s intrinsic properties into holography, unlocking a scalable and secure paradigm for ultrahigh-dimensional information technologies.

## Abstract (web summary)

We establish operator-enabled holography as a new paradigm, transcending intrinsic physical dimensions of light to achieve high-capacity and high-security multiplexing.

## Graphical abstract

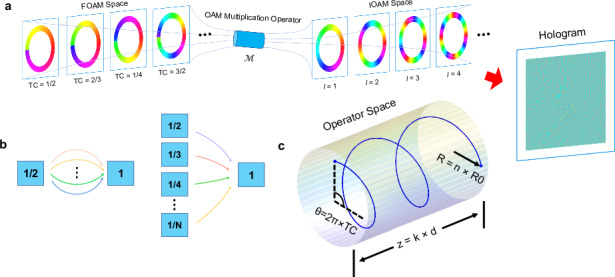

## Introduction

Optical holography is a technique introduced by Dennis Gabor in 1948 for the optimization of electron microscopy^1,2^, which consists of the recording and reconstruction process based on the interference of multiple wave fields. Thanks to its ability to record both amplitude and phase information, holography technology has become a potentially powerful tool for the general public to realize the reconstruction of custom three-dimensional (3D) objects, especially with the rapid development of computer science and computer-generated holograms (CGHs)^3,4^. CGH enables the simulation of interference recording directly on a computer, bypassing the need for optical setups. It has been widely used in 3D display^5–7^, image projection^8^, beam shaping^9,10^, ultrashort pulse laser parallel processing^11–13^ and nonlinear holography^14,15^. To enhance the storage capacity of a single hologram, various physical degrees of freedom of light, including time^16^, polarization^17–19^, and wavelength^20,21^, are systematically exploited in the design process to generate a hologram capable of multiplexing multiple datasets. Orbital angular momentum (OAM), an additional physical degree of freedom, is manifested by a spiral phase structure *exp*(*ilϕ*) where *l* is the topological charge and *ϕ* is the azimuthal angle. OAM beam has garnered significant research interest due to its diverse potential applications, encompassing optical tweezers^22,23^, optical communications^24,25^, and quantum information processing^26–28^, largely attributed to their theoretically unbounded helical mode index. Recently, this unique physical degree of freedom has been leveraged in holography to unlock new possibilities in data encryption^29–35^ and optical information storage^36–39^. OAM holography has also been successfully implemented in the nonlinear optics regime^40,41^.

Multiplexed holography has achieved remarkable progress by systematically exploiting the intrinsic physical dimensions of light. However, the extensive utilization of these native parameters leaves minimal scope for advancing holographic methodologies. To unlock new frontiers, it is essential to transcend conventional frameworks by engineering new dimensions beyond the physical properties of light. Operator, defined as a function over a space of physical states onto another space of states, offers a transformative framework.

**Fig. 1:**
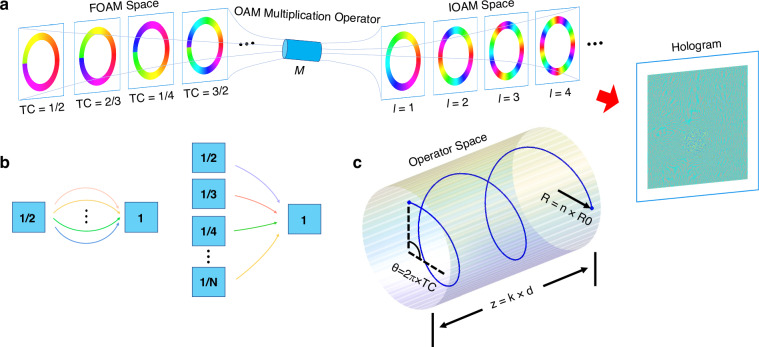
Concept of OAM operator enabled holography. **a** Schematic of the optical OAM multiplication operator ${\mathscr{M}}$, mapping FOAM modes to IOAM modes for holographic reconstruction. **b** Non-unique operator pathways. A FOAM mode (TC = 1/2) can map to an IOAM mode (TC = 1) via distinct ${\mathscr{M}}$-pathways, while an IOAM mode (TC = 1) can also be generated from multiple FOAM modes (e.g., TC = 1/2, 1/3 and 1/4). **c** Operator parametric space representation. Each ${\mathscr{M}}$-pathway (e.g., TC = 9/4, *n* = 4, *d* = 1.6 cm) maps to a unique helical trajectory, where TC is the topological charge of the incident fractional OAM beam, *n* is the multiplication scaling factor and *d* is the propagation distance, respectively in the method of coordinate transformation

In this work, we propose and experimentally demonstrate an operator-enabled holographic multiplexing framework. Here, we use an optical OAM multiplication operator as an example. By mapping fractional OAM (FOAM) modes in FOAM space to integer OAM (IOAM) modes in IOAM space through predefined transformation pathways (Fig. 1a), we realize holographic reconstruction using the generated IOAM modes. This process is defined by:

1 $${\mathcal{M}}| {\psi }_{{\rm{FOAM}}}\left.\right\rangle =| {\psi }_{{\rm{IOAM}}}\left.\right\rangle \mathop{\longrightarrow}\limits^{{\rm{reconstruct}}}H$$

where ${\mathcal{M}}$ represents the multiplication operator, and *H* denotes the OAM hologram. Notably, a single IOAM mode can originate from multiple distinct FOAM modes through different operator mappings, while the operator itself is non-unique for a given FOAM-to-IOAM transformation (Fig. 1b). We further construct an operator-specific hologram that exclusively responds to the pre-defined operator pathway. We also demonstrate the orthogonality between distinct operator pathways. Consequently, an operator-multiplexed hologram encoding through distinct pathways can be designed. This work offers a novel approach to OAM holography with high security, high capacity and high fidelity, demonstrating significant potential for applications in the field of cryptographic systems, information storage, and dynamic 3D displays. More fundamentally, it introduces the concept of operators as an additional dimension into the holographic system. This approach breaks the dependence of conventional multiplexed methodologies on the intrinsic physical dimensions of light. Such operator-enabled holography pioneers a paradigm shift, laying the groundwork for next-generation holographic technology.

## Results

### Implementation of the optical OAM multiplication operator

**Fig. 2:**
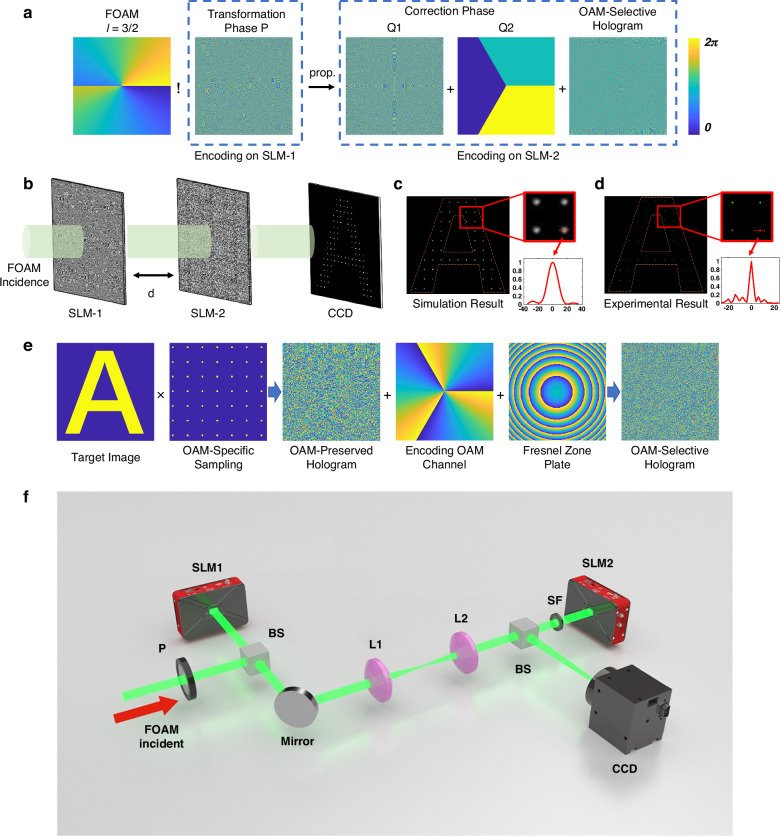
Implementation of optical OAM multiplication operator. **a** Principle of coordinate transformation for OAM multiplication. **b** Schematic diagram of the optical holographic system. **c** Simulation and **d** experimental results when the target image is Letter A and parameters are set to be TC = 3/2, *n* = 2, *d* = 1 cm and *l* = −3. **e** Construction of OAM-selective hologram. **f** Schematic of the experimental setup (see Methods for more details)

The optical OAM multiplication operator is realized through the method of coordinate transformation^42,43^, as illustrated in Fig. 2a. Its implementation relies on the transformation phase *P* applied at the input plane, a specific propagation distance *d*, and the correction phases *Q* (including two parts: *Q*1 and *Q*2) incorporated at the output plane (see Supplementary Note 1 for details):

2 $$P(r,\theta )=\frac{k}{d}\left[\frac{c{r}^{1-1/n}}{1-1/n}\cos \left(\theta -\frac{\theta }{n}\right)-\frac{{r}^{2}}{2}\right]$$

3 $${Q}_{1}(\rho ,\varphi )=-P-k\sqrt{{r}^{2}+{\rho }^{2}-2r\rho \cos (\varphi -\theta )+{d}^{2}}$$

4 $${Q}_{2}(\rho ,\varphi )=2\pi t\left[\frac{n\varphi }{2\pi }\right]$$

To verify the theory, we first use the resulting IOAM mode to reconstruct the target image from an OAM-selective hologram. The design principle of the OAM-selective hologram is depicted in Fig. 1e. According to the OAM-matching condition, the target image can be reconstructed only when the condition TC × *n* + *l* = 0 is satisfied, where *n* is the multiplication scaling factor, TC and *l* are the topological charge of the incident FOAM beam and the encoded OAM channel, respectively. To simplify the optical setup, the transformation phase *P* is loaded onto SLM_1, while the correction phase *Q* and the OAM-selective hologram are loaded onto SLM_2, as shown in Fig. 1b. The distance between SLM_1 and SLM_2 corresponds to the propagation distance *d* in the coordinate transformation process. As a straightforward example, we set the topological charge of the incident FOAM beam TC = 3/2, transformation scaling factor *n* = 2, and propagation distance *d* = 1 cm. The Letter A is encoded onto the OAM channel with *l* = −3. The simulation and experimental results are shown in Fig. 2c, d. The emergence of a pixelated Letter A, along with the Gaussian shape of each pixel, demonstrates that the FOAM mode (TC = 3/2) has been successfully transformed into an IOAM mode (TC = 3) and validates the effectiveness of the holographic reconstruction through the multiplication operator. Further details on the efficiency of OAM multiplication are provided in Supplementary Note 2.

**Fig. 3:**
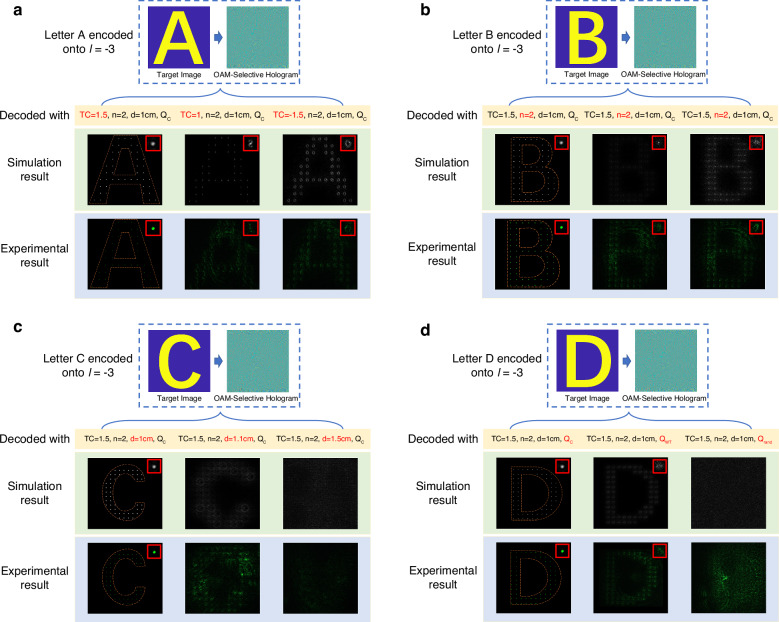
Four influencing factors of OAM operator enabled holography. **a** Topological charge of the incident FOAM beam TC. Reconstructed images under TC deviations exhibit distortion (e.g., s-shape for TC = 1 and doughnut-shape for TC = −3/2) compared to the Gaussian-shape pixels at TC = 3/2. **b** Transformation scaling factor *n*. Using mismatched values of *n* (e.g., *n* = 3 or *n* = 4) degrades each pixel into low-intensity and noise-like distribution. **c** Propagation distance *d*. A minor offset of Δ*d* = 0.1 cm renders the letter ''C” unrecognizable, while Δ*d* = 0.5 cm erases all structural information. **d** Correction phase *Q*. Substituting the correct phase *Q*_*c*_ (designed for TC = 3/2, *n* = 2, *d* = 1 cm) with mismatched *Q*_wr_ (TC = 4/3, *n* = 3, *d* = 1 cm) or random *Q*_rand_ phases leads to blurred or fully scrambled speckle patterns, respectively. Typical intensity profiles of individual pixels under each parameter condition are labeled

Crucially, the precise synchronization of four parameters governs the fidelity of FOAM-to-IOAM conversion, thereby directly determining the feasibility of reconstructing target holographic images. To investigate the specific influence of the parameter TC, we encode the Letter A in the OAM channel of *l* = −3 and examine three scenarios. In the first scenario, the multiplication mode (TC = 3/2, *n* = 2, *d* = 1 cm, *Q*_*c*_) is applied, with *Q*_*c*_ as the corresponding correction phase, under which the target image is reconstructed. In the second scenario, while keeping *n*, *d*, *Q*_*c*_ unchanged, TC is reset to 1. In the third scenario, TC is set to the opposite of the first case, with all other parameters unchanged. The simulation and experimental results are presented in Fig. 3a. When TC = 1, the coordinate transformation introduces an OAM mismatch Δ*l* = 1, compounded by an incorrect correction phase. As a result, each pixel in the reconstructed image exhibits a s-shape low-intensity distribution. For TC = –3/2, the correction phase *Q* is identical for FOAM beams with topological charges of 3/2 and –3/2. Consequently, each pixel in the reconstructed image manifests as a vortex ring, corresponding to an OAM mismatch of Δ*l* = 6. The simulation results clearly demonstrate that patterns arising from incorrect TC can be interpreted as noise, particularly after applying specific post-processing techniques^33^. However, the experimental results present more ambiguity. The primary cause of this phenomenon is experimental imperfections in the optical path, leading to defects in the IOAM beams generated through coordinate transformation. Even with a correct TC, the reconstructed Gaussian-shaped pixels exhibit residual noise around their periphery. When OAM mismatch or correction phase deviations occur, the pixel distributions become significantly broader and more complex. These effects, illustrated in Supplementary Note 3, result in pronounced interference between adjacent pixels, producing the blurred patterns observed in Fig. 3a. In our experiments, the sampling constant was set to 20*λ*/NA, which preserves the Gaussian-shaped central pixels while suppressing sidelobe interference.

Similarly, we encode the letters B, C, and D in the OAM channel of *l* = −3. When the mode (TC = 3/2, *n* = 2, *d* = 1 cm, *Q*_*c*_) is applied, the target image is successfully reconstructed in all cases. To investigate the effect of the transformation scaling factor *n*, we separately set *n* to 3 and 4. Under these conditions, each pixel degrades from a high-intensity Gaussian shape to a low-intensity speckled pattern, as shown in Fig. 3b. The deviation in propagation distance *d* has an even more detrimental effect on the reconstructed image. As seen in Fig. 3c, a 0.1 cm error renders the letter C unrecognizable. When the error reaches 0.5 cm, no meaningful information can be extracted from the reconstructed result. For the correction phase *Q*, we replace the correct correction phase *Q*_*c*_(corresponding to the multiplication process with TC = 3/2, *n* = 2, and *d* = 1 cm) with a wrong correction phase *Q*_*wr*_(corresponding to the multiplication process with TC = 4/3, *n* = 3, and *d* = 1 cm), as well as with a random phase *Q*_*rand*_. The experimental and simulation results are shown in Fig. 2d. In the case of *Q*_*wr*_, the image becomes blurred and indistinguishable. In the case of *Q*_*rand*_, the image completely degrades into random speckles.

**Fig. 4:**
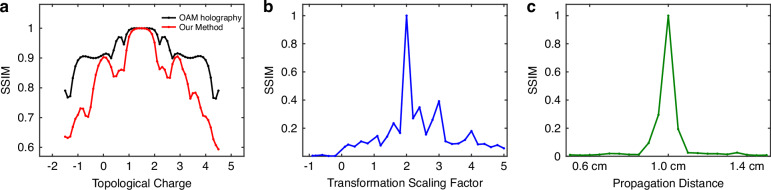
Parameter sensitivity analysis. **a** Structural similarity index (SSIM) degradation under fractional topological charge TC offset. Operator-enabled holography exhibits faster SSIM decline (red curve) compared to conventional OAM holography (black curve) cross all offset ranges. Even minor deviations in the **b** transformation scaling factor *n* and **c** propagation distance *d* result in dramatic SSIM drops. Narrow bandwidth highlights the stringent requirement for the parameter accuracy

We further evaluated the structural similarity index (SSIM) under varying parameter offsets. Under precise parameter alignment, the result exhibits high SSIM, demonstrating the high-fidelity reconstruction capability. When offset exists, the SSIM decline will be induced. Specifically, as shown in Fig. 4a, the deviation of the incident topological charge ΔTC leads to a more rapid SSIM decline than conventional OAM holography across all offset ranges, owing to the mismatch with other transformation parameters. Notably, even minor deviations in the transformation scaling factor and propagation distance result in a dramatic SSIM drop (Fig. 3b, c), highlighting the stringent requirement for parameter accuracy. These largely suppress the crosstalk between neighboring channels and enable high multiplexing density in subsequent implementations. Representative reconstructed images for different offsets are provided in Supplementary Note 4.

### ${\mathcal{M}}$-specific hologram

**Fig. 5:**
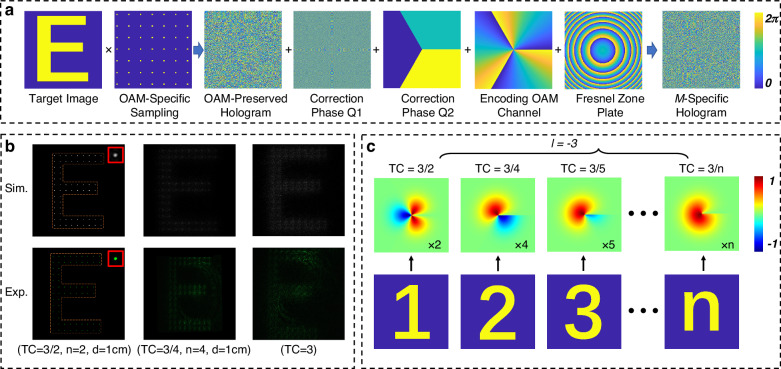
${\mathcal{M}}$-specific holography. **a** Design of a ${\mathcal{M}}$-specific hologram by adding the correction phase into the OAM channel. **b** Reconstruction specificity. Experimental results confirm that only the designated operator pathway ${\mathcal{M}}$(TC = 3/2, *n* = 2, *d* = 1 cm) reconstructs the target image (e.g., ''E''), while alternative processes fail. The decoding multiplication modes are listed at the bottom. **c** Single OAM channel subdivided into operator-defined sub-channels, each acting as an independent information carrier

In “Implementation of the optical OAM multiplication operator”, the design of holograms and multiplication operators have been treated as separate processes. A given IOAM channel can be reconstructed through multiple operator pathways or even direct IOAM incidence, as the holographic channels lack intrinsic specificity to the operator. This independence introduces critical security vulnerabilities. To address this limitation, we propose a ${\mathcal{M}}$-specific hologram by directly encoding the correction phase (associated with a designated operator condition) into the OAM channel. As shown in Fig. 5a, the correction phase corresponding to ${\mathcal{M}}$(TC = 3/2, *n* = 2, *d* = 1 cm) was directly encoded onto an OAM-selective hologram (*l* = −3), leading to a ${\mathcal{M}}$-specific hologram. This ensures that only the predefined operator pathway can reconstruct the target image (e.g., letter “E” in Fig. 5b). Competing operators, such as (TC = 3/4, *n* = 4, *d* = 1 cm) or IOAM incidence, fail due to phase mismatch, as validated experimentally.

To establish operator as a dimension for holographic multiplexing, certain orthogonality between distinct operator pathways must be guaranteed. Through theoretical derivation (see Supplementary Note 5 for details), we derive the orthogonality condition for two operator pathways:

5 $$\left\langle ({\text{TC}}_{1},{n}_{1},{d}_{1})\left\vert ({\text{TC}}_{2},{n}_{2},{d}_{2})\right.\right\rangle ={\delta }_{{{\rm{TC}}}_{1},{\text{TC}}_{2}}\cdot {\delta }_{{n}_{1},{n}_{2}}\cdot {\delta }_{{d}_{1},{d}_{2}}$$

To geometrically characterize each operator pathway ${\mathcal{M}}$(TC, *n*, *d*), we can map each unique combination of TC, *n*, and *d* to a distinct helical trajectory in a 3D parametric space (Fig. 1c). These trajectories are mathematically described by:

6 $$\left\{\begin{array}{l}x=n{R}_{0}\cos (2\pi \cdot \,\text{TC}\,\cdot s)\quad \\ y=n{R}_{0}\sin (2\pi \cdot \,\text{TC}\,\cdot s)\quad \\ z={k}_{z}\cdot d\cdot s\quad \end{array}\right.$$

where *k*_*z*_ governs the axial scaling of propagation distance *d*, *R*_0_ defines the base radius, and *s* ∈ [0, 1] parameterizes the normalized progression along the pathway. Consequently, the accumulated phase *θ* = 2*π* ⋅ TC dictates the parameter of TC, while the radius *R* = *nR*_0_ and the axial displacement *z*_*max*_ = *k*_*z*_ ⋅ *d* explicitly encode *n* and *d*, respectively. This geometric mapping ensures a one-to-one correspondence between operators and their helical representations, enabling intuitive visualization of parameter interdependencies.

From an information capacity perspective, traditional OAM holography faces a fundamental trade-off: while the theoretically unbounded helical modes promise unlimited channels, higher-order modes demand larger sampling intervals to resolve spatial-frequency components, degrading resolution and constraining practical capacity. Our approach circumvents this bottleneck by subdividing a single OAM channel into multiple orthogonal ${\mathcal{M}}$-based sub-channels. As illustrated in Fig. 5c, the IOAM channel *l* = −3 can be partitioned into sub-channels governed by fractional TC values (e.g., TC = 3/2, 3/4, 3/5) and scaling factors (e.g., *n* = 2, 4, 5). Each sub-channel operates as an independent information carrier enabling parallel data encoding.

### ${\mathcal{M}}$-multiplexed hologram

**Fig. 6:**
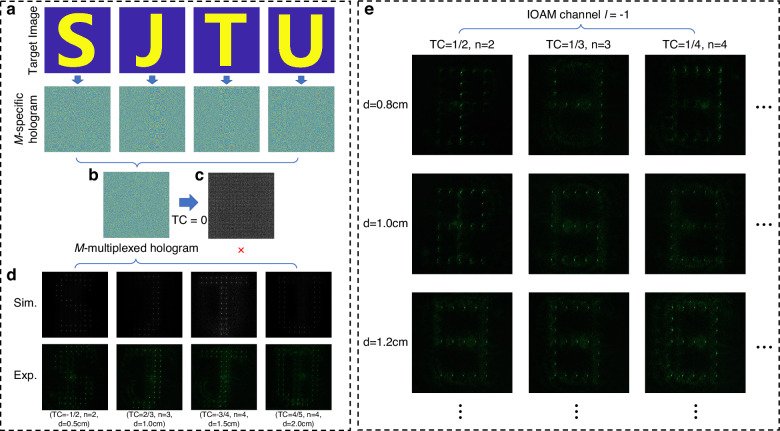
${\mathcal{M}}$-multiplexed holography for ultrahigh-capacity encoding. **a** The design approach for a ${\mathcal{M}}$-multiplexed hologram. **b** A multiplexed hologram containing the information of the letters “SJTU''. **c** Planar wave reconstruction. No discernible information is retrieved under planar wave illumination. **d** Simulation and experimental reconstruction with the correct operator pathways. **e** 9-channel ${\mathcal{M}}$-multiplexed hologram, achieving ninefold capacity enhancement over conventional OAM holography

Based on the strong ${\mathcal{M}}$-selectivity, ${\mathcal{M}}$-multiplexation can be achieved to encode multiple holographic images into one single hologram. The multiplexing approach is illustrated in Fig. 6a. The letters “SJTU” were selected as the target image. Each of the four letters was first independently encoded into four OAM channels (*l* = 1, −2, 3, −4), following the method illustrated in Fig. 2c, resulting in four OAM-selected holograms. Subsequently, these four holograms were superimposed with different correction phase modes, corresponding to the operators of ${\mathcal{M}}$(TC = −1/2, *n* = 2, *d* = 0.5 cm), ${\mathcal{M}}$(TC = 2/3, *n* = 3, *d* = 1.0 cm), ${\mathcal{M}}$(TC = −3/4, *n* = 4, *d* = 1.5 cm) and ${\mathcal{M}}$(TC = 4/5, *n* = 5, *d* = 2.0 cm), respectively. This process generated four ${\mathcal{M}}$-specific holograms, which were then combined to construct the final ${\mathcal{M}}$-multiplexed hologram (Fig. 6b). It is confirmed that planar wave illumination fails to reconstruct any meaningful information (Fig. 6c), whereas sequential illumination with the designated operator pathway selectively reconstructs each target image with minimal crosstalk with an average signal-to-noise ratio (SNR) of 24.51 dB for simulation results and 18.96 dB for experimental results (Fig. 6d), after applying an intensity thresholding method (detailed in “Methods”) to further improve the image quality.

To assess the capacity enhancement of our ${\mathcal{M}}$-multiplexed framework, we assigned three TC-*n* pairs (TC = 1/2, *n* = 2), (TC = 1/3, *n* = 3) and (TC = 1/4, *n* = 4) to three distinct *d* values (0.8 cm, 1.0 cm, 1.2 cm), collectively defining nine ${\mathcal{M}}$-channels. Adjacent channels maintain a minimum separation of Δ*d* = 0.2 cm (for identical TC-*n* pairs) or Δ*n* = 1 (for identical *d*), ensuring the minimal crosstalk between arbitrary two channels as demonstrated by the sensitivity analysis in Fig. 4b, c. The nine digits of Arabic numerals 1 to 9 were encoded into these channels to construct a 9-channel multiplexed hologram. Experimental reconstructions (Fig. 6e) exhibit high-quality reconstruction of all 9 images (simulation details in Supplementary Note 6). Crucially, nine channels leverage respective ${\mathcal{M}}$-pathway to generate an IOAM mode (TC = 1) for decoding the target image encoded in the *l* = −1 channel, thereby expanding the capacity of a single OAM channel by a factor of 9. This ninefold increase does not represent the intrinsic upper limit of our method, but rather the constraint imposed by the use of phase-only holograms^44^.

### ${\mathcal{M}}$-multiplexed holography for high-security encryption

**Fig. 7:**
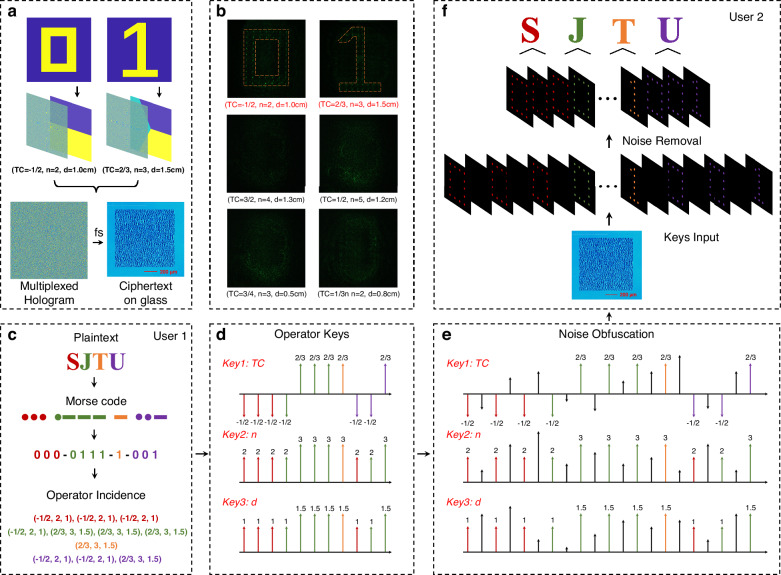
High-security encryption via ${\mathcal{M}}$-multiplexed holography. **a** Design of the ${\mathcal{M}}$-multiplexed hologram. Binary patterns ''0” and ''1” are encoded into distinct operator pathways.The hologram is binarized using a modified off-axis encoding method and fabricated inside glass via femtosecond laser writing. **b** Experimental reconstruction. Correct parameters reconstruct “0” and “1'', while deviations yield no discernible patterns. **c** Plaintext-to-ciphertext conversion. Plaintext “SJTU” is converted to Morse code (“0” for dots, “1” for dashes) and mapped to sequential operator pathways. **d** Synchronized keychain generation. Three parameter keys (TC, *n*, *d*) are generated, each strictly aligned with the binary sequence. **e** Noise obfuscation. Random values are injected into the keychains to disrupt deterministic parameter correlations while preserving valid sequence alignment. **f** Decryption workflow. User2 applies obfuscated keychains to reconstruct the hologram, filters noise via intensity thresholding, and decodes the Morse sequence to recover “SJTU''

To validate the ultra-secure encryption capability of the proposed ${\mathcal{M}}$-multiplexed holography, we implemented a hierarchical encryption framework that synergizes ${\mathcal{M}}$-channel multiplexing, synchronized keychains, and adaptive noise obfuscation. As depicted in Fig. 7b, the encryption process begins with the conversion of the plaintext “SJTU” into a binary Morse sequence (dots as “0”, dashes as “1”), which is subsequently mapped to two distinct operator pathways: “0” corresponds to ${\mathcal{M}}$(TC = −1/2, *n* = 2, *d* = 1.0 cm), while “1” is assigned to ${\mathcal{M}}$(TC = 2/3, *n* = 3, *d* = 1.5 cm). Then, a 2-bit ${\mathcal{M}}$-multiplexed hologram was constructed and binarized through a modified off-axis binary encoding method (Supplementary Note 7). The hologram was inscribed into a glass substrate via femtosecond laser writing, forming a physical ciphertext resistant to tampering (Fig. 7a).

The binary sequence generates three synchronized keychains (TC, *n*, *d*), where each parameter set strictly aligns with the predefined operator pathways (Fig. 7d). Crucially, the original keychains exhibit a deterministic structure—each parameter chain contains only two values corresponding to “0” and “1,” with identical value transitions across all chains. This structural predictability poses a critical security risk, as attackers could exploit the synchronized parameter variations to infer the encryption logic. To address this vulnerability, random yet structurally compatible parameter values were injected into the keychains while preserving the spatial and logical alignment of valid parameters (Fig. 7e). This noise obfuscation ensures that unauthorized reconstruction yields only stochastic speckle patterns, whereas legitimate users exploit the synchronized keychains to isolate valid binary sequences through intensity thresholding and spatial filtering. Experimental validation shown in Fig. 7b confirms that correct parameter sets reconstruct sharp “0” and “1” patterns, while others result in unrecognizable intensity distributions.

The decryption workflow requires User2 to sequentially illuminate the ciphertext along the operator pathways matching the obfuscated keychains. A noise removal protocol leveraging intensity thresholding filters out invalid reconstructions. The final Morse code is translated back to the plaintext “SJTU” (Fig. 7f), demonstrating end-to-end information fidelity.

## Discussion

This work proposes an optical operator-enabled holography framework based on the multiplication of FOAM modes, offering a novel approach to holographic encoding and multiplexing. Our design ensures that each holographic channel responds exclusively to the specific operator pathway. The construction of ${\mathcal{M}}$-multiplexed holograms further demonstrates a novel approach to high-capacity information storage and encryption. The demonstrated ninefold capacity enhancement represents only a conservative proof of concept. Theoretically, each IOAM channel can be subdivided into an unbounded number of operator-defined sub-channels, as the parametric space of operator offers infinite orthogonal channels. To further enhance the performance of operator-enabled holography, our strategy can be combined with other advanced holographic techniques. For capacity enhancement, complex-amplitude metasurfaces, which enable accurate convolution between complex-amplitude image channels and OAM helical wavefronts, can enable ultrahigh-capacity multiplexing^44^. For resolution improvement, time-division multiplexing^37^ offers a powerful approach by converting coherent interference into incoherent accumulation, thereby mitigating the resolution loss imposed by sampling requirements. These techniques are fully compatible with our operator-enabled framework, offering exciting opportunities for future developments.

Traditional OAM holography permits partial information leakage even with incorrect topological charges, as mismatched modes still generate recognizable patterns such as doughnut-shaped intensity distributions. In contrast, the proposed operator-based OAM holography eliminates such weakness through stringent interdependence among the four critical parameters. For instance, a deviation as small as Δ*d* = 0.1 cm reduces the reconstructed image to noise-like distributions. This extreme sensitivity to parameter alignment ensures that only precise combinations yield meaningful reconstructions, effectively preventing unauthorized decryption. To further demonstrate the robustness of our approach under practical non-idealities, we conducted additional simulations on radial-mode contamination, as presented in Supplementary Note 5, confirming that the operator-enabled framework maintains strong orthogonality even in such non-ideal condition. Furthermore, the method’s robustness extends to applications like 3D holographic displays, as shown in Supplementary Fig. 11. We present the same two target images encoded with the OAM channels and ${\mathcal{M}}$-channels, with the imaging depth determined by the focal length of Fresnel Zone Plates. Traditional 3D OAM holography exhibits inherent crosstalk, where OAM-mismatched reconstruction produces doughnut-shaped patterns, thereby affecting the corresponding image formation. While in 3D operator-enabled holography, mismatched modes dissipate into unstructured speckles rather than forming low-intensity doughnut-shaped recognizable patterns, minimizing cross-plane interference without requiring additional spatial filters.

In conclusion, we introduce the concept of operator as a synthetic dimension beyond the intrinsic physical degrees of freedom of light, pioneering a novel paradigm for multiplexed holography. While the OAM multiplication operator serves as a foundational example, OAM division operators^43^ and other potential optical operators^45^ may similarly integrate with holographic systems. The operator-enabled framework demonstrates a universal platform for ultrahigh-capacity information storage and high-security cryptographic methodologies, establishing a foundation for next-generation optical holographic data storage systems, dynamic holographic displays, and secure optical encryption architectures.

## Method

### Experimental setup

The experimental setup is illustrated in Fig. 2f. The light source is a continuous wave (CW) laser with the wavelength of 532 nm. The polarization of the FOAM beam is changed to horizontal direction by using the polarizer (P). Subsequently, the FOAM beam is reflected by SLM1, which is loaded with the transformation phase map. The SLM we use is a reflective phase-only liquid crystal modulator (UPOLabs, HDSLM80R), which has a rate of 60 Hz and a resolution of 1920 × 1080 pixels. The input light is illuminated onto SLM2 loaded with the correction phase and the OAM-selective hologram via a 4f system composed of lenses L1 and L2. The focal lengths of the lenses used in the experiment are all *f* = 100 mm. There is a distance of f+d between SLM2 and L2, which ensures the free propagation of light between the input and output plane at a distance of d. A spectral filtering process is implemented via a spatial filter (SF) to isolate the desired holographic pattern. Finally, the holographic pattern is imaged by a CMOS camera (CCD) at the imaging depth.

### Fabrication of CGH by femtosecond laser erasure

The CGH is fabricated using a NIR laser working at 800 nm wavelength, 1 kHz reputation rate, and 109 fs pulse width (Coherent Legend Elite). A fused silica glass (eagle XG) with a thickness of 1 mm is used as a sample which is mounted on the computer-controlled XYZ translation stage with a resolution of 0.2 μm. An objective lens with a numerical aperture of 0.75 (CFI Plan Fluor 40X) is applied to focus the laser pulse onto the surface of the crystal. The moving speed is 100 μm/s and the moving direction is perpendicular to the laser beam. In our experiment, a single pulse of about 50 μJ is used. Controlled by the computer program, the lattice structure of the glass is selectively destroyed corresponding to the dark area of the CGHs. While the non-irradiated points correspond to the white area of the CGHs. In this case, the decrement of the refrective index is large enough, so that the structure can be approximately seen to be opaque^46^. We fabricated the CGH patterns with 450 × 450 pixels, where each pixel has a size approximately at 2 μm × 2 μm. The fabricated CGHs were written within an area of 0.9 × 0.9 mm^2^ and the total processing time is 3 h.

### Signal-to-noise ratio (SNR) calculation and intensity thresholding

We need to quantitatively evaluate the quality of reconstruction and the crosstalk between distinct operator pathways in the ${\mathcal{M}}$-multiplexed holography. To this purpose, the averaged SNR was defined as:

7 $$\,{\text{SNR (dB)}}\,=10{\log }_{10}\left(\frac{{I}_{{\rm{signal}}}}{{I}_{{\rm{noise}}}}\right)$$

where *I*_signal_ and *I*_noise_ represent the integration intensity of holographic images reconstructed from the desired ${\mathcal{M}}$-channel and other multiplexing channels, respectively.

To suppress noise in experimental reconstructions, an intensity thresholding method is applied. The threshold value *T* is set to 10% of the maximum pixel intensity ${I}_{\max }$ within the reconstructed image ($T=0.1\times {I}_{\max }$). Pixels with values below *T* are truncated to zero, while values above *T* remain unchanged. The thresholded image is visually inspected to ensure critical features. The SNR calculation and thresholding are implemented via a custom MATLAB script.

## Supplementary information

Supplementary Information for OAM Multiplication Operator Enabled Holographic Multiplexing

## Data Availability

The data that supports the results within this paper and other findings of the study are available from the corresponding authors upon reasonable request.

## Code availability

The custom code and mathematical algorithm used to obtain the results within this paper are available from the corresponding authors upon reasonable request.
