# Supplementary material for: OAM multiplication operator enabled holographic multiplexing
Source: Light Sci Appl. 2026 Jan 2;15:18. doi: 10.1038/s41377-025-02107-2 (PMC12757602; doi:10.1038/s41377-025-02107-2)
Supplement: Supplementary file 1 — Supplementary Information for OAM Multiplication Operator Enabled Holographic Multiplexing [file 41377_2025_2107_MOESM1_ESM.pdf]

Supplementary Information for
OAM Multiplication Operator Enabled Holographic Multiplexing

Feiyang Shen[1,†], Zhengyang Mao[1,†], Weiwen Fan[2,†], Jiangwei Wu[1], Zhifan Fang[1], Haigang Liu[1,*], Xianfeng Chen[1,3,*], Yong Zhang[2,*] and Yuping Chen[1,*]

[1]*School of Physics and Astronomy, State Key Laboratory of Photonics and Communications, Shanghai Jiao Tong University, 800 Dongchuan Road, Shanghai 200240, China*

[2]*National Laboratory of Solid State Microstructures, College of Engineering and Applied Sciences, Nanjing University, Nanjing 210093, China*

[3]*Collaborative Innovation Center of Light Manipulations and Applications, Shandong Normal University, Jinan 250358, China*

Feiyang Shen: shenfeiyang828@sjtu.edu.cn
Zhengyang Mao: single_dm@sjtu.edu.cn
Weiwen Fan: fww18021319944@163.com
Jiangwei Wu: wjw2016@sjtu.edu.cn
Zhifan Fang: fang20011220@sjtu.edu.cn
Haigang Liu: liuhaigang@sjtu.edu.cn*
Xianfeng Chen: xfchen@sjtu.edu.cn*
Yong Zhang: zhangyong@nju.edu.cn*
Yuping Chen: ypchen@sjtu.edu.cn*
School of Physics and Astronomy, Shanghai Jiao Tong University,
800 Dongchuan Road, Shanghai 200240, China
Tel: +86-13816373910
College of Engineering and Applied Sciences, Nanjing University,
163 Xianlin Road, Nanjing 210093, China
Tel: +86-13951851980

[†] *These authors contributed equally to this work.*

Supplementary Note 1: Principle of Coordinate Transformation for OAM multiplication

The coordinate transformation theory in ray optics facilitates precise mapping operations for the spatial positions of light. Considering the paraxial propagation of a collimated light beam between two planes separated by a distance d — with the input plane defined by coordinates (x, y) and the output plane by (u, v) — the generalized Snell's law establishes a coordinate transformation between corresponding points on the two planes, expressed as [1]:

$$P_x = k\frac{u - x}{d}, \quad P_y = k\frac{v - y}{d} \tag{S1}$$

where k represents the wave vector in free space, and $P(x, y)$ denotes the transformation phase applied at the input plane. By modulating $P(x, y)$ appropriately, various coordinate transformations can be achieved. A spiral transformation is a distinctive case that maps one spiral geometry to another. In this transformation, the input and output planes are redefined using spiral-polar coordinates, characterized by a radial coordinate r and a spiral azimuthal coordinate varying over the range $[0, +\infty)$. Unlike conventional polar coordinates, this approach imposes no 2π restriction on angular coordinates, enabling a finer and more continuous partitioning of the plane.

The transformation laws governing spiral transformation are defined as:

$$\rho = cr^{-1/n}, \quad \varphi = \frac{\theta}{n} \tag{S2}$$

where (r, θ) and (ρ, φ) denote the coordinates of the input and output planes, respectively. The parameter c is an arbitrary constant, and n represents the transformation factor. Assuming that (r, θ) lies on a spiral defined by $r = ae^{b\theta}$, Equation (2) implies that (ρ, φ) will similarly reside on a new spiral expressed as $\rho = ca^{-1/n}e^{-b\varphi}$. Using Equations (1) and (2), the transformation phase $P(x, y)$ required on the input plane can be determined as:

$$P(r,\theta)=\frac{k}{d}\left[\frac{cr^{1-1/n}}{1-1/n}\cos\left(\theta-\frac{\theta}{n}\right)-\frac{r^2}{2}\right] \tag{S3}$$

Another phase mask $Q_1(x,y)$ (correction phase) on the output plane is required to compensate for both $P(x,y)$ and the propagation phase generated during the propagation in free space, which is written as:

$$Q_1(\rho,\varphi)=-P-k\sqrt{r^2+\rho^2-2r\rho\cos(\varphi-\theta)+d^2} \tag{S4}$$

For FOAM input modes, an additional correction phase Q_2 is superimposed onto Q_1, which is expressed as:

$$Q_2(\rho,\varphi)=2\pi t\left[\frac{n\varphi}{2\pi}\right] \tag{S5}$$

where $t=mod(l_{\mathrm{in}},1)$. Such a requirement stems from the unique characteristic of the FOAM modes—its phase distribution in the azimuthal direction is discontinuous [2].

The FOAM optical field can be expressed as $E_1(r,\theta)=R(r)exp(il_{\mathrm{in}}\theta)$ where $R(r)$ represents the relationship between the amplitude of FOAM and the radius r in polar coordinates, and l_{in} is a noninteger topological charge. After the transformation and the phase correction, the field E_2 at the output point (ρ,φ) is equal to the field E1 at the corresponding input point (r,θ) multiplied by a factor, which is given by

$$E_2(\rho,\varphi)=-iexp(ikd)\left|n\right|\frac{r}{\rho}E_1(r,\theta) \tag{S6}$$

The relationship between (r,θ) and (ρ,φ) is given by Equation (2). Therefore, the exponential term in Equation (6) is $exp(il_{\mathrm{in}}\theta)=exp(inl_{\mathrm{in}}\varphi)$, which indicates that the transformed beam is a vortex beam carrying OAM with $l_{\mathrm{out}}=nl_{\mathrm{in}}$.

Supplementary Note 2: Efficiency of OAM Multiplication based on Coordinate Transformation.

In the OAM multiplication process, the key step is the geometric coordinate transformation that rescales the azimuthal angle as

$$\phi' = n\phi \tag{S7}$$

where n is the multiplication scaling factor.

This rescaling modifies the azimuthal period of the optical field and redistributes its power. The overlap between the transformed field and the corresponding integer OAM (IOAM) eigenmode determines the fraction of optical power remaining in the desired channel. For OAM multiplication with $|n| > 1$, only a fraction

$$\eta_{\text{theory}} = \frac{1}{|n|} \tag{S8}$$

with the remainder distributed into sidebands.

In experiments, the achieved efficiency is typically lower due to alignment imperfections, mode mismatch, and optical losses. Importantly, in our framework the efficiency is always sufficient for the reconstructed Gaussian-shaped pixels to stand well above the residual background. This robustness benefits from the strong orthogonality between different operator pathways, where unmatched channels contribute only weak background-like noise (see Figure 3 in the main text). As a result, reliable image retrieval is ensured even when the absolute efficiency is below the theoretical bound.

In the future, the efficiency can be further improved by enhancing system stability and alignment. In addition, metasurface-based complex-amplitude holography or alternative operator implementations may provide new routes toward higher efficiency in future platforms.

Supplementary Note 3: Pixel distribution under OAM matching and mismatching condition.

In conventional OAM sorting systems, when the topological charge of the input vortex beam matches the charge of the compensating phase plate, the resulting beam collapses into a Gaussian-like central spot because the OAM phase term is canceled, leaving a planar wavefront. Conversely, when a mismatch exists, the residual OAM generates a doughnut-shape intensity profile. The radius of this ring scales linearly with Δl, as governed by the Fourier relationship between azimuthal phase gradients and radial intensity modulations.

In our scheme, the coordinate transformation process introduces subtle wavefront distortions due to the experimental imperfections in the optical path. This modifies the pixel intensity distributions even under OAM-matched conditions. The output exhibits a central Gaussian-like core but is surrounded by faint concentric rings and azimuthally fragmented sidelobes. These features arise from non-ideal alignment of the coordinate transformation optics. Under mismatched conditions, the intensity distribution retains a doughnut-shaped profile at its center, but with a serrated edge rather than an ideal annular symmetry. Additionally, complex sidelobe structures surround the central doughnut-shaped region. The experimental results of pixel intensity distributions for both OAM matching and mismatching cases can be found with detailed characterization in our prior work [2]. Importantly, the mode distributions under both OAM matching and mismatching conditions exhibit significantly larger spatial footprints compared to those in conventional OAM holography. To preserve the FOAM characteristics during reconstruction, the sampling interval must be designed to exceed the spatial extent of the individual pixel mode distribution. This requirement leads to a larger sampling interval in our method compared to traditional OAM holography, inherently limiting the native resolution of the holographic image. To overcome this trade-off, the method of temporal multiplexing can be applied to suppresses the adjacent pixel interference (API) [3], thereby achieving higher holographic imaging resolution.

Supplementary Note 4: Impact of parameter deviations on holographic image reconstruction.

The structural similarity index (SSIM) quantifies the perceptual difference between a reconstructed image I and the target image T. It combines luminance, contrast, and structural comparisons, defined as:

$$\mathrm{SSIM}(I,T) = \frac{2\mu_I\mu_T + C_1}{\mu_I^2 + \mu_T^2 + C_1} \cdot \frac{2\sigma_{IT} + C_2}{\sigma_I^2 + \sigma_T^2 + C_2} \tag{S9}$$

where μ_I and μ_T are the mean intensities, σ_I^2 and σ_T^2 are the variances, σ_{IT} are the covariance, and $C_1 = (k_1L)^2, \quad C_2 = (k_2L)^2$ c $(k_1 = 0.01, k_2 = 0.03, L = 1)$. Values closer to 1 indicate higher similarity.

The target image was encoded into the operator pathway ($TC = 3/2, n = 2, d = 1\,cm$). Here, we demonstrate the reconstructed images under systematic parameter offsets, as shown in Fig. S1-S3.

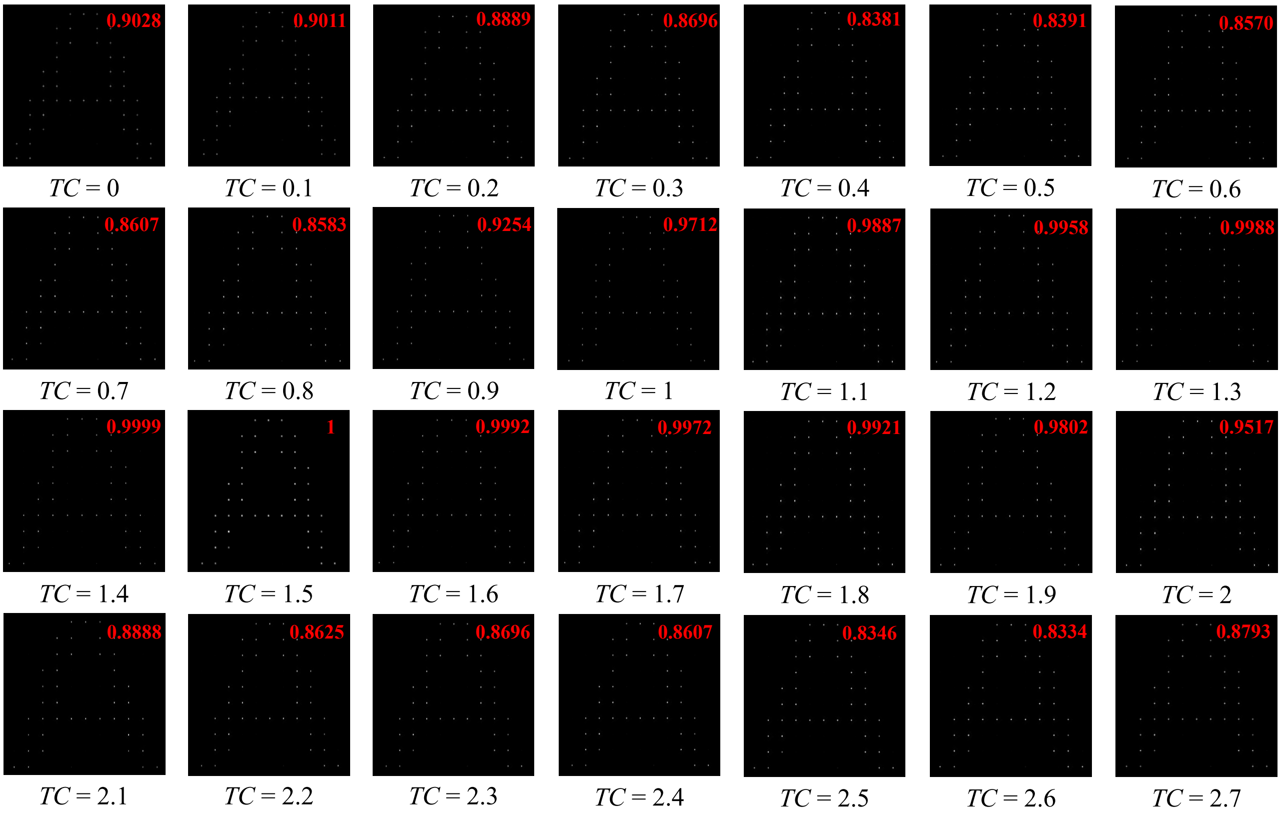

Figure S1: Reconstructed images when topological charge TC offsets (incident TC ranging from 0 to 2.7, step= 0.1). The SSIM values are labeled.

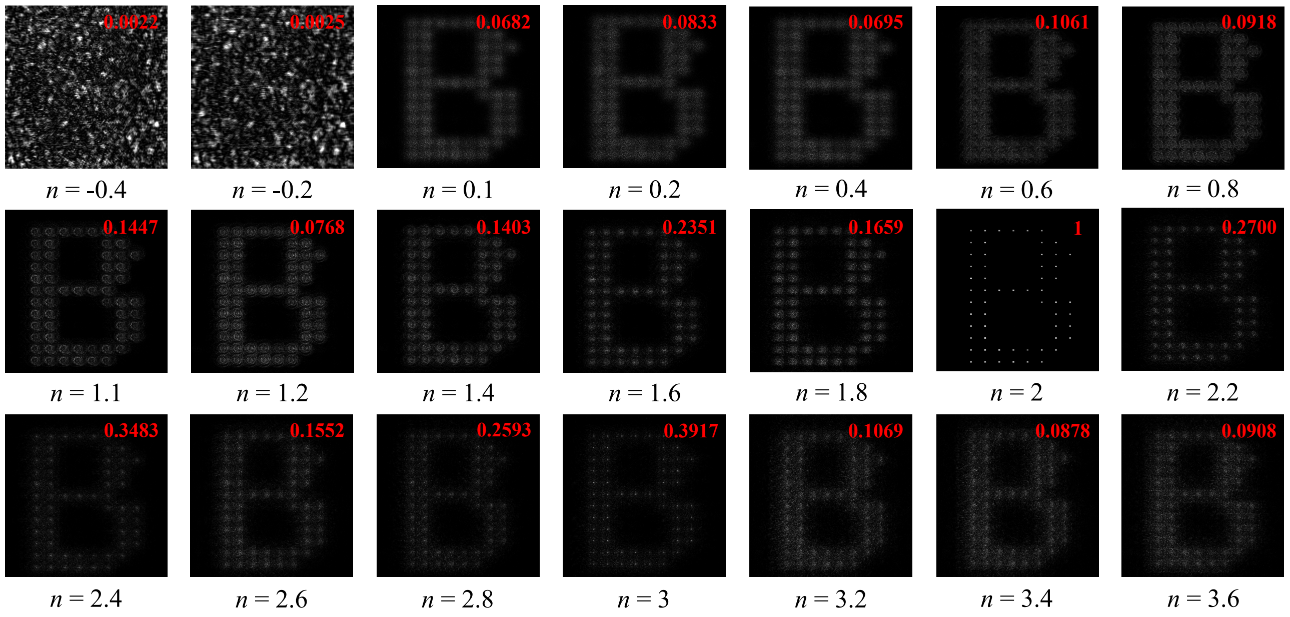

Figure S2: Reconstructed images under different scaling factor n (n ranging from -0.4 to 3.6, step$=0.2$, and invalid transformation at $n = 0$ and $n = 1$ are excluded). The SSIM values are labeled.

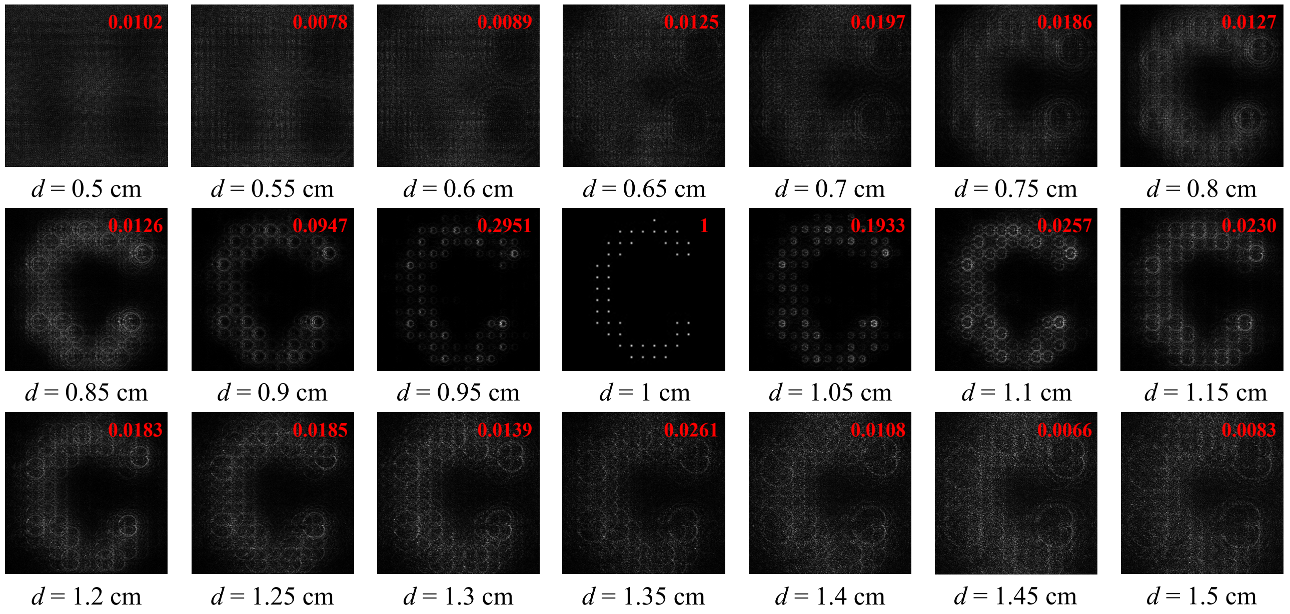

Figure S3: Reconstructed images under different propagation distance d (d ranging from 0.5 cm to 1.5 cm, step=0.05 cm). The SSIM values are labeled.

Supplementary Note 5: The orthogonality of two operator pathways.

To establish operators as synthetic dimensions in holographic multiplexing, we rigorously analyze the orthogonality between distinct operator pathways $\mathcal{M}(TC, n, d)$. The orthogonality condition ensures the independence between operator channels and forms the foundation of ultrahigh-dimensional encoding. Below, we detail the mathematical derivation and numerical validation of this critical property.

Under the action of an operator, the complex amplitude obtained on the output plane after the input light field undergoes a transformation phase P and propagates a distance d is:

$$E_{\text{out}}(\rho,\varphi) = \text{C}\cdot R\left(\left(\frac{c}{\rho}\right)^n\right)\cdot\exp\left(\text{i}\, TC\cdot n\cdot\varphi\right)\cdot\exp\left(\text{i}\, P\left(\left(\frac{c}{\rho}\right)^n, n\varphi\right)\right)\cdot\exp\left(\text{i}\,\Phi_{\text{prop}}(\rho,\varphi)\right) \tag{S10}$$

where P is the transformation phase and $\Phi_{\text{prop}}(\rho,\varphi)$ is the propagation phase corresponding to the distance d:

$$\Phi_{\text{prop}}(\rho,\varphi) = k\sqrt{r^2+\rho^2-2r\rho\cos(\varphi-\theta)+d^2} \tag{S11}$$

Consider two operator pathways:

Operator 1: (TC_1, n_1, d_1), output field $E_{out}^{(1)}$;

Operator 2: (TC_2, n_2, d_2), output field $E_{out}^{(2)}$;

The inner product between these pathways is defined as:

$$\left\langle E_{\text{out}}^{(1)}\middle| E_{\text{out}}^{(2)}\right\rangle = \iint\limits_{\rho,\varphi} A(\rho)\,\exp\left[i\left(\Delta l\cdot\varphi + \Delta P(\rho,\varphi) + \Delta\Phi_{\text{prop}}(\rho,\varphi)\right)\right]\rho\, d\rho\, d\varphi \tag{S12}$$

where:

- $\Delta l = n_1\cdot TC_1 - n_2\cdot TC_2$
- $\Delta P = P_1 - P_2$
- $\Delta\Phi_{\text{prop}} = \Phi_{\text{prop}}^{(1)} - \Phi_{\text{prop}}^{(2)}$
- $A(\rho) = R_1(r)\cdot R_2(r)$, where $r = \left(\frac{c}{\rho}\right)^n$

The First Term: Δl

When $\Delta l \neq 0$, this term introduces a rapidly oscillating phase factor with respect to φ over the interval $[0, 2\pi]$, resulting in:

$$\int_0^{2\pi} e^{i\Delta l\varphi}\, d\varphi = 0 \tag{S13}$$

In practice, the other two phase terms, $\Delta P(\rho, \varphi)$ and $\Delta\Phi_{\text{prop}}(\rho, \varphi)$, also depend on φ. However, these dependencies are typically smooth and slowly varying. As such, they act as modulation functions and the oscillatory nature of the first term dominates the angular integration. Therefore, the integration of the first phase term behaves like a Kronecker delta function and can be effectively factored out of the double integral. The overall inner product between two operator pathways becomes:

$$\big\langle (TC_1, n_1, d_1) \big| (TC_2, n_2, d_2) \big\rangle \approx \delta_{n_1 \cdot TC_1, n_2 \cdot TC_2} \cdot \iint A(\rho)\, e^{i\,\Delta P(\rho,\varphi)}\, e^{i\,\Delta\Phi_{\text{prop}}(\rho,\varphi)}\, \rho\, d\rho\, d\varphi \tag{S14}$$

The Second Term: ΔP

The second exponential term in the overlap integral,

$$e^{i\Delta P(\rho,\varphi)} = e^{i(P_1(\rho,\varphi) - P_2(\rho,\varphi))} \tag{S15}$$

originates from the coordinate transformation phase applied to the input plane. According to Eq. (S3), this transformation phase depends nonlinearly on both the transformation scaling factor n and the propagation distance d. Even when the azimuthal mismatch term vanishes ($\Delta l = 0$), a difference in n or d causes $\Delta P(\rho, \varphi)$ to exhibit spatially varying phase profiles. This non-negligible phase deviation leads to destructive interference across the integration domain and significantly reduces the overlap integral. Therefore, the overall inner product between two operator pathways can be further expressed as:

$$\big\langle (TC_1, n_1, d_1) \big| (TC_2, n_2, d_2) \big\rangle \approx \delta_{n_1 \cdot TC_1, n_2 \cdot TC_2} \cdot \delta_{n_1, n_2} \cdot \delta_{d_1, d_2} \cdot \iint A(\rho)\, e^{i\,\Delta\Phi_{\text{prop}}(\rho,\varphi)}\, \rho\, d\rho\, d\varphi \tag{S16}$$

The Third Term: $\Delta\Phi_{\text{prop}}$

The third exponential term in the overlap integral,

$$e^{i\Delta\Phi_{\text{prop}}(\rho,\varphi)} = e^{i\left(\Phi^{(1)}_{\text{prop}}(\rho,\varphi)-\Phi^{(2)}_{\text{prop}}(\rho,\varphi)\right)} \tag{S17}$$

arises from the accumulated phase during free-space propagation over different distances d_1 and d_2. According to Eq. (S8), a small deviation in propagation distance introduces a nonlinear phase mismatch due to the square-root dependence on d. This mismatch modulates the wavefront curvature across the output plane, resulting in rapidly varying phase differences over the spatial domain. As a result, this phase term imposes a similarly strict constraint on the equality of d_1 and d_2, effectively acting as a Kronecker delta condition with respect to the propagation distance. Finally, the orthogonality condition between two multiplication operator pathway is derived:

$$\big\langle (TC_1, n_1, d_1) \big| (TC_2, n_2, d_2) \big\rangle \approx \delta_{TC_1,TC_2} \cdot \delta_{n_1,n_2} \cdot \delta_{d_1,d_2} \tag{S18}$$

To validate the effectiveness and correctness of the derived orthogonality condition, we performed numerical simulations of Eq. (S9) by systematically calculating the normalized inner products under certain deviations of $TC, n\ and\ d$, as illustrated in Figs. 1-3. For comparative analysis, we also simulated the conventional orbital angular momentum orthogonality condition (Fig. 4), which is fundamentally governed by the azimuthal phase orthogonality:

$$\langle l_1 | l_2 \rangle = \int_0^{2\pi} e^{i(l_2-l_1)\phi}\, d\phi = 2\pi\delta_{l_1,l_2} \tag{S19}$$

The results demonstrate rigorous agreement with the proposed orthogonality framework, validating the correctness of the orthogonality condition.

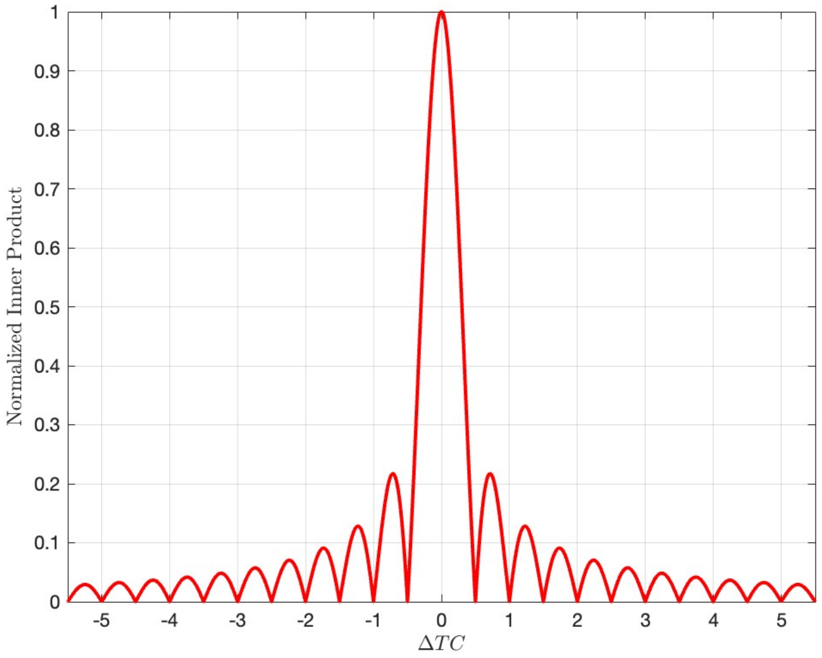

Figure S4: Normalized inner product between two operator pathways as a function of topological charge deviation.

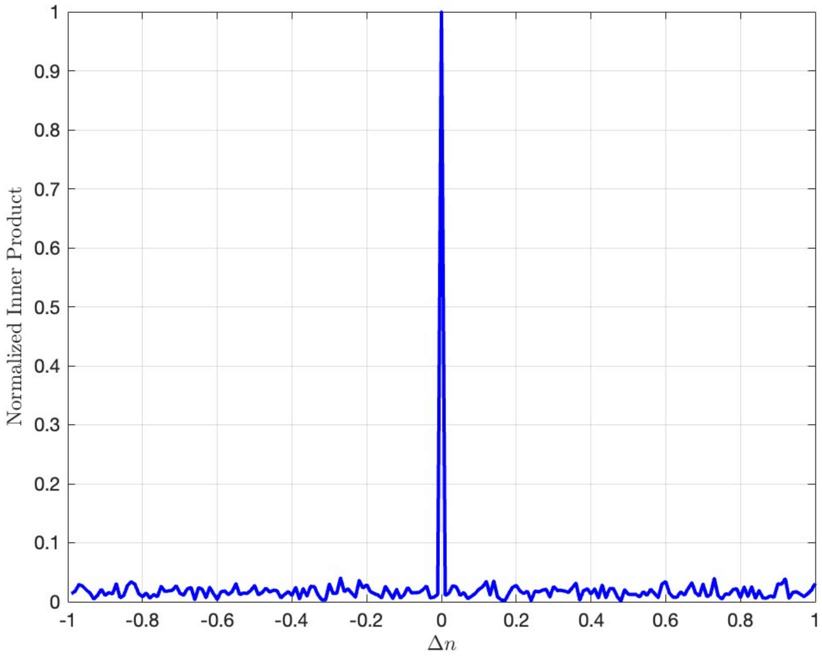

Figure S5: Normalized inner product between two operator pathways as a function of transformation scaling factor deviation.

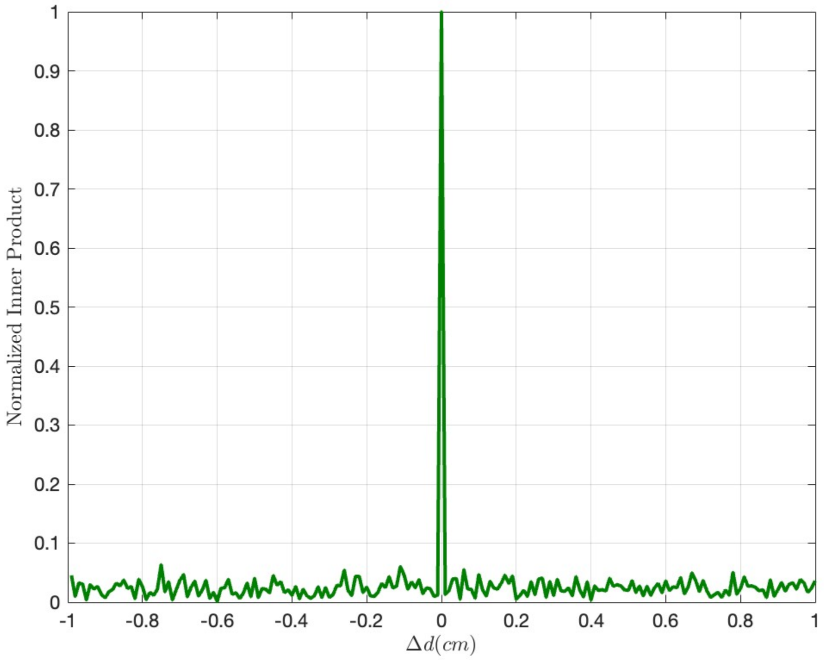

Figure S6: Normalized inner product between two operator pathways as a function of propagation distance deviation.

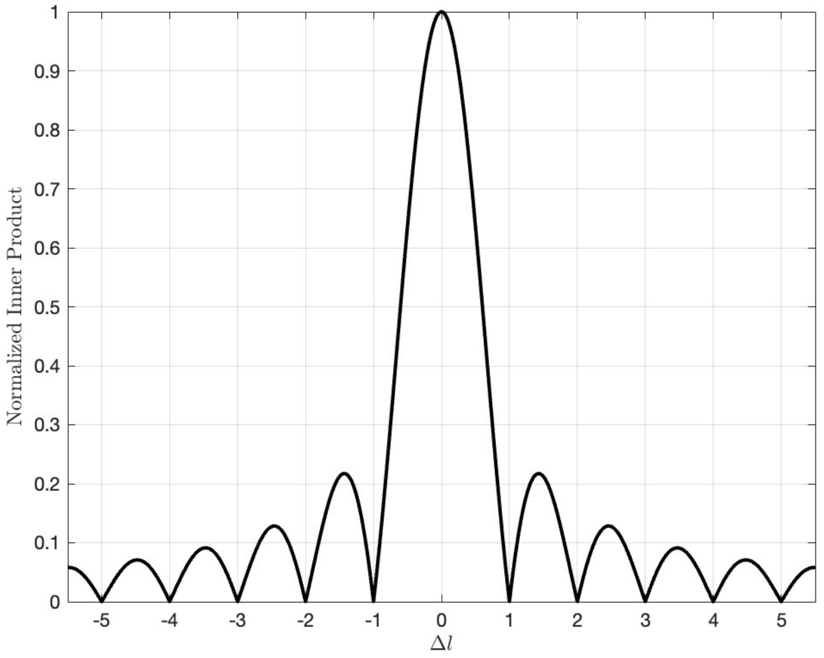

Figure S7: Normalized inner product between two conventional OAM modes as a function of topological charge deviation.

Robustness Analysis

To evaluate the robustness of the orthogonality between operator pathways un-

der realistic experimental conditions, we investigate the impact of radial mode contamination, a common imperfection arising from scattered light higher order mode excitation or imperfect beam shaping. We intentionally introduce radial mode contamination by modifying the ideal Perfect Vortex beam envelope. The contaminated optical field is described by

$$E(r,\theta) = \left[\exp\left(-\frac{(r-R_0)^2}{W^2}\right) + 0.5\exp\left(-\frac{r^2}{R_0^2}\right) + 0.3\exp\left(-\frac{(r-1.6R_0)^2}{(1.2W)^2}\right)\right] e^{i\theta} \tag{S20}$$

where the three terms represent the ideal main vortex ring, a strong Gaussian background and a prominent secondary vortex ring, respectively. This model effectively captures typical experimental imperfections.

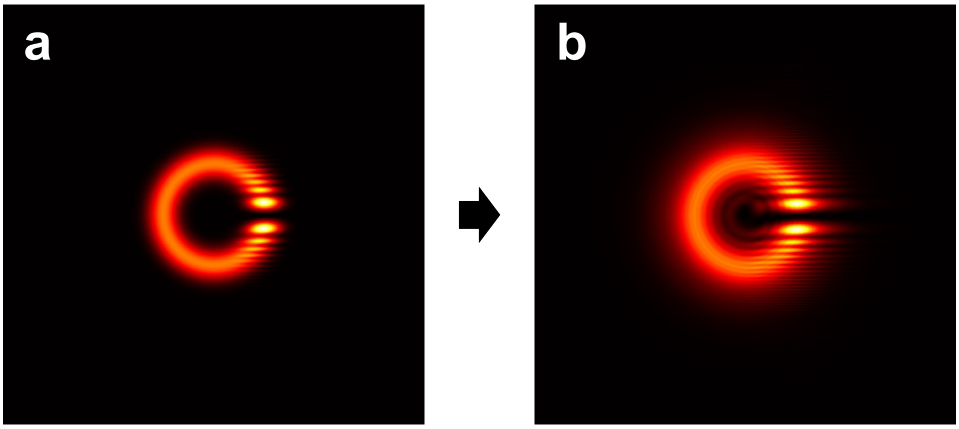

Figure S8: Intensity distribution of (a) ideal FOAM beam ($TC = 1.5$) and (b) radial-mode contaminated FOAM Beam.

Despite this substantial contamination altering the radial intensity distribution as shown in Figure S8, the inner product between two operator pathways still exhibits sharp Dirac-Delta-like peaks. Figure S9 confirms that orthogonality is well maintained even under such non-ideal conditions.

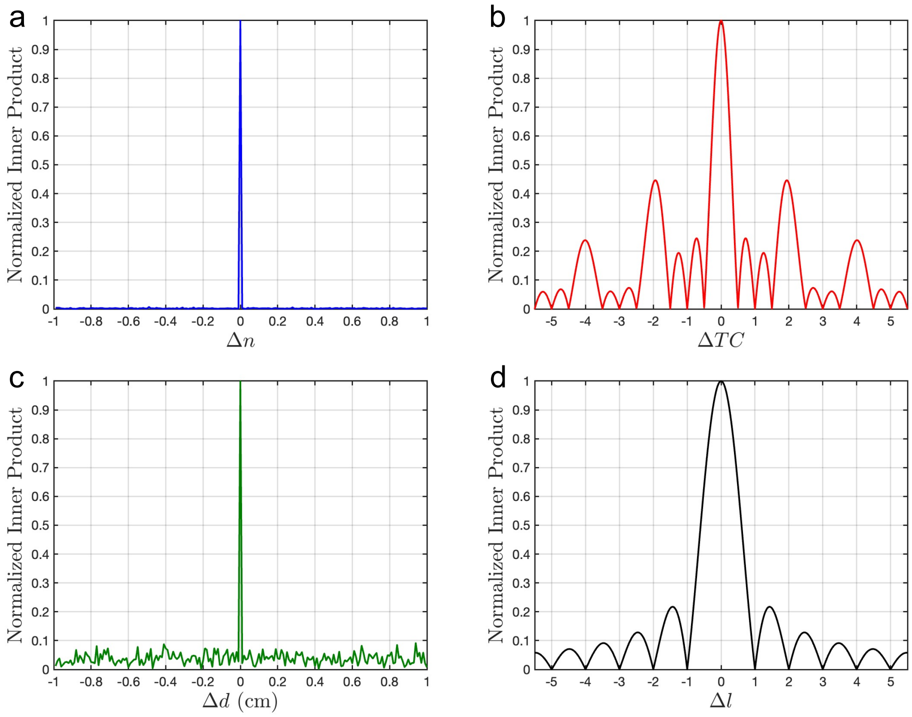

Figure S9: Normalized inner product between two operator pathways considering the effect of radial-mode contamination as a function of (a) transformation scaling factor deviation, (b) topological charge deviation and (c) propagation distance deviation. (d) Normalized inner product between two conventional OAM modes under the same condition is also provided.

This robustness analysis strongly suggests that common experimental imperfections, such as slight misalignment in the optical path or deviations from an ideal beam profile, are unlikely to compromise the orthogonality between distinct multiplication operator pathways, thereby affirming the practical feasibility and reliability of our proposed multiplexing scheme.

Supplementary Note 6: Simulation of high-capacity multiplexed hologram.

To validate the feasibility of our 9-channel operator-multiplexed hologram, numerical simulations were conducted by encoding the nine digits of Arabic numerals 1 to 9 into distinct orthogonal operator channels. Each channel corresponds to a unique combination of parameters: three TC-n pairs (TC=1/2, n=2; TC=1/3, n=3; TC=1/4, n=4) and three propagation distances ($d = 0.8, 1.0, 1.2$). The hologram was synthesized by superimposing nine OAM-selective sub-holograms, each generated using the Gerchberg-Saxton algorithm with a corresponding helical phase correction ($l = -1$) and Fresnel zone plate. The reconstructed images (Fig. S8) demonstrate the successful reconstruction of all 9 Arabic numerals.

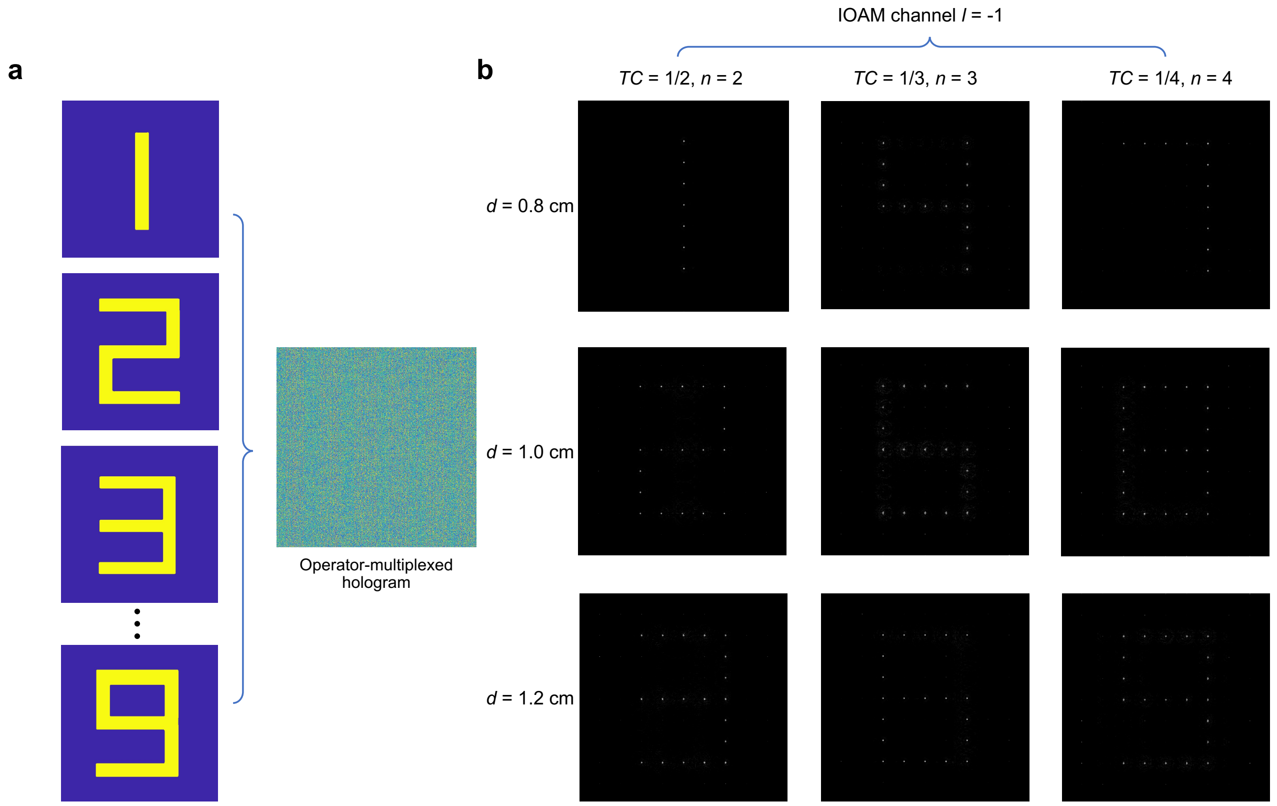

Figure S10: Simulation of 9-channel operator-multiplexed hologram. (a) Schematic of the multiplexed hologram design. Numerical labels 1–9 are encoded into 9 orthogonal channels. (b) Simulated reconstruction results for all 9 channels.

Supplementary Note 7: Binarization of the complex-amplitude hologram.

The complex-amplitude multiplexed hologram, encoding both amplitude and phase, is binarized using an off-axis interference encoding scheme. This method ensures that the binary hologram retains sufficient information to reconstruct $H(x, y)$ under coherent illumination. The hologram is modulated by a tilted plane wave with spatial carrier frequency, generating a real-valued interference pattern:

$$H_{\text{offaxis}}(x, y) = \text{Re}\left[H(x, y) \cdot e^{i2\pi\alpha y}\right] = \frac{1}{2}H(x, y)e^{i2\pi\alpha y} + \frac{1}{2}H^*(x, y)e^{-i2\pi\alpha y}. \tag{S21}$$

Binarization is performed by thresholding this pattern into two levels:

$$CGH_{\text{binary}}(x, y) = \begin{cases} 1, & \text{if } H_{\text{offaxis}}(x, y) \geq 0, \\ 0, & \text{otherwise.} \end{cases} \tag{S22}$$

The binary hologram CGH_{binary} approximates the interference structure of H_{offaxis}, acting as a high-contrast amplitude grating. When illuminated by a collimated plane wave $U_{\text{in}}(x, y) = 1$, the transmitted field becomes:

$$U_{\text{out}}(x, y) \approx \frac{1}{2} + \frac{1}{2}H(x, y)e^{i2\pi\alpha y} + \frac{1}{2}H^*(x, y)e^{-i2\pi\alpha y}. \tag{S23}$$

The second term encodes the original complex field $H(x, y)$, modulated by the spatial carrier $e^{-i2\pi\alpha y}$. This term diffracts at an angle $\theta = arcsin(\lambda\alpha)$. The third term contains the conjugate field of $H(x, y)$, propagating symmetrically to the second term. While the first term represents the undiffracted light propagating along the optical axis, carrying no object information.

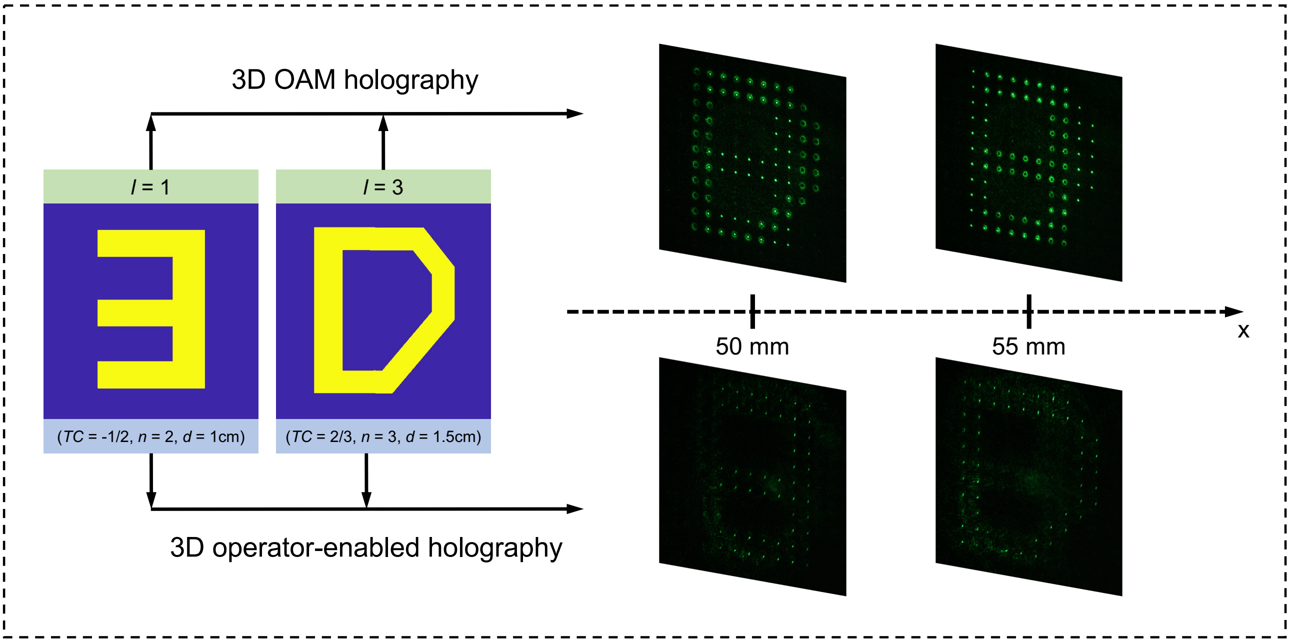

Figure S11: 3D holographic reconstruction comparison between traditional and operator-enabled implementations. The target images “3” (imaging depth: $50\,mm$) and “D” ($55\,mm$) are simultaneously encoded through traditional OAM channels ($l = 1$ and $l = 3$) and operator channels with $\mathcal{M}(TC = -1/2, n = 2, d = 1\,cm)$ and $\mathcal{M}(TC = 2/3, n = 3, d = 1.5\,cm)$. Experimental results demonstrate that operator-mismatched modes degrade into unstructured speckles, whereas OAM-mismatched modes retain recognizable patterns.

References

[1] Yoshiharu Saito, Shin-ichi Komatsu, and Hitoshi Ohzu. Scale and rotation invariant real time optical correlator using computer generated hologram. Optics Communications, 47(1):8–11, 1983.

[2] Zhengyang Mao, Haigang Liu, and Xianfeng Chen. Effective sorting of fractional optical vortex modes. Advanced Photonics Nexus, 3(6):066001–066001, 2024.

[3] Zijian Shi, Zhensong Wan, Ziyu Zhan, Kaige Liu, Qiang Liu, and Xing Fu. Super-resolution orbital angular momentum holography. Nature Communications, 14(1):1869, 2023.
